# A Nonspatial Sound Modulates Processing of Visual Distractors in a Flanker Task

**DOI:** 10.3758/s13414-020-02161-5

**Published:** 2020-10-20

**Authors:** Cailey A. Salagovic, Carly J. Leonard

**Affiliations:** University of Colorado Denver, Psychology Department

## Abstract

Successful navigation of information-rich, multimodal environments involves processing of both auditory and visual information. The extent to which information within each modality is processed varies due to many factors, but the influence of auditory stimuli on the processing of visual stimuli in these multimodal environments is not well understood. Previous research has shown that a preceding sound leads to decreased reaction times in visual tasks (Bertelson, 1967). The current study examines if a non-spatial, task-irrelevant sound additionally alters processing of visual distractors that flank a central target. We utilized a version of a flanker task in which participants responded to a central letter surrounded by two irrelevant flanker letters. When these flankers are associated with a conflicting response, a congruency effect occurs such that reaction time to the target is slowed (Eriksen & Eriksen, 1974). In two experiments using this task, results showed that a preceding tone caused general speeding of reaction time across flanker types, consistent with alerting. The tone also caused decreased variation in response time. Critically, the tone modulated the congruency effect, with a greater speeding for congruent flankers than for incongruent flankers. This suggests that the influence of flanker identity was more intense after tone presentation, consistent with a nonspatial sound increasing perceptual and/or response-association processing of flanking stimuli.

Processing sensory information is vital for normal functioning, however the immense amount of input that the brain receives at any one time cannot all be processed to the same degree. Generally, attention functions to prioritize a subset of stimuli from the environment to be processed further, but information from task-irrelevant stimuli also has a strong influence on the system. Studies have shown evidence against a strict model of discrete processing stages gated by an early locus of attentional selection (*e.g*. Moore & Egeth, 1997; Vogel, Luck, & Shapiro, 1998). Early support for this idea came from Erik Eriksen and colleagues, who proposed a ‘continuous flow’ model of processing in which information accumulation in the visual system about the target may occur in concurrence with response associations about distractors (Eriksen & Schultz, 1979). Given that humans are adapted to live in environments with simultaneous input from multiple sensory modalities, it is of particular interest how this continuous flow of processing is influenced by information integrated across these different sources.

Although a great deal of research has examined how visual attention influences information processing, the extent to which auditory stimuli can interact with this information processing remains unclear. Despite the relative lack of research, several fundamental audiovisual interactions are generally accepted. In real-world environments, sound commonly accompanies visual events and has an effect on visual attention. Perhaps the most robust interaction is the orienting effect of spatially-localized sounds. When a sound can be discerned as having come from a specific location, such as the beeping of a car horn on the road, attention may be allocated to that area (Hillyard, Störmer, Feng, Martinez, & McDonald, 2016), enhancing processing of visual input (Frassinetti, Bolognini, & Làdavas, 2002). As with visual objects, attention also tends to be directed to sounds that provide information related to a current goal (Fritz, Elhilali, David, & Shamma, 2007). Additionally, sounds can direct visual attention to semantically congruent stimuli (Mastroberardino, Santangelo, & Macaluso, 2015). For example, the whistling of a kettle signifying the need to turn off the heat, can attract attention to the stovetop. However, not all sounds are spatial or task-relevant. With the ubiquity of technology such as earbuds and music streaming services, experiencing sounds which are non-spatial and not relevant to current-task performance is becoming increasingly common. Furthermore, these sounds are likely completely independent of the visual scene.

Previous research has shown that the onset of a non-spatial, irrelevant auditory stimulus decreases reaction time (RT) to subsequent visual stimuli (Bertelson, 1967). These results are consistent with the activation of an alerting network which may serve to prime the motor system in a generalized manner to execute responses faster (*e.g*., Niemi & Näätänen, 1981). Posner and Petersen (1990) proposed that alerting does not influence the build-up of visual information, but rather allows more rapid response selection. However, in addition to providing this type of alerting effect, sound may also alter the continuous flow of how visual stimuli are processed during perception or response-selection processes. Experiments have demonstrated that a task-irrelevant, non-spatial sound occurring temporally near the second target in a rapid serial visual presentation (RSVP) stream significantly increased identification of that target (Chen & Yeh, 2008). In particular support of the view that sound alters the spatial allocation of visual attention, Kusnir, Chica, Mitsumasu, and Bartolomeo (2011) found that a preceding sound improved detection of a near-threshold visual target presented at one of two possible peripheral locations. However, none of these studies evaluated the effect of auditory stimuli on the processing of visual stimuli at task-irrelevant locations.

One method that has traditionally been used to assess the processing of irrelevant information in the visual field is the flanker task developed by Eriksen & Eriksen (1974). This task presents a central target flanked on each side by one or more additional distractors. Participants are asked to respond to the identity of the central target while disregarding the task-irrelevant flankers. When the response assigned to the central target does not match that of the flankers, RT is slowed due to processing of this incompatible response. This indicates that despite the target always occurring at fixation, information from flanking distractors is processed to the level of response to some degree. This flanker compatibility effect can be modulated by various factors including flanker eccentricity, attentional focus, and perceptual load (Miller, 1991).

Previous research has shown that the influence of flankers is modulated by the degree to which attention is allocated to them. Generally, when a perceptually demanding stimulus requires focused attention at a location, sensitivity to more peripheral stimuli is decreased (Carmel, Thorne, Rees, & Lavie, 2011). In contrast, increasing attentional allocation to flanking stimuli can enhance the degree to which they are integrated into perceptual processing (Freeman, Sagi, & Driver, 2001). More specifically, response-congruency effects from flankers have been shown to increase under conditions that promote attention to flankers (Gaspelin, Ruthruff, & Jung, 2014). Thus, the increase in attention to peripheral locations caused by sound, as purported by Kusnir *et al.* (2011), may be predicted to cause a similar increase in congruency effects.

In the current study, we used a flanker task with letter stimuli to specifically examine how a nonspatial auditory tone may change the processing of task-irrelevant distractors. Consistent with the literature on alerting (Bertelson, 1967), we expected that the tone would cause general speeding of RT to subsequently-presented visual stimuli. Given the literature discussed above showing that a task-irrelevant nonspatial sound may change attentional allocation, we hypothesized that such a sound may alter attention to the flankers. The zoom lens model proposed by Eriksen and St. James (1986) is a useful framework through which to consider how a nonspatial sound may change attention. They propose that the scope of spatial attention is flexible, such that it can range from narrowly focused to more broadly distributed. In the case of a flanker task, a narrow focus of attention might be more optimal. However, if the spatial distribution of attention were to be expanded, flankers may be processed to a greater extent leading to an increased effect of congruency. Although this attentional theory is motivated by previous literature, it must also be noted that increased interference from incongruent flankers would also be consistent with the tone influencing later stages of the continuous flow of information processing. This is further considered in Experiment 2.

## Experiment 1

### Methods

#### Participants

A total of 18 participants completed Experiment 1 and received course credit, and 3 were excluded due to technical difficulties with task presentation or eye-tracking data collection. Fifteen participants (mean age 19.7, 14 female) were included in the analysis reported below, which was the planned-N chosen to be comparable with that used in previous investigations of flanker effect modulation (Eriksen & Eriksen, 1974; Chen &Yeh, 2008; Weinbach & Henik, 2012). All participants were recruited through the University of Colorado Denver participant pool and earned course credit for participation. Eligible participants reported having normal or corrected-to-normal vision and hearing, and no neurological impairments. Before beginning the experiment, all participants gave written informed consent. IRB approval for this study was obtained from the University of Colorado Denver COMIRB.

#### Apparatus and Stimuli

Participants were seated at a desk in a dimly lit room and instructed to place their chin in a chin rest situated 80 cm away from a 24-inch monitor. During the experiment, eye movements were recorded by an SR Research 1000 Plus desk-mounted eye tracker linked to the experimental computer via Matlab and Psychtoolbox software (Brainard, 1997). The eye tracker was calibrated to the participant’s right eye before beginning the experiment and during the task as necessary. Eye-tracking was done only as a means to ensure central fixation at the beginning of each trial and there was no a priori plan to investigate eye movements^1^. Participants wore Audio-technica ATH-ANC20 headphones and used a Logitech videogame controller to make responses. The participant was overseen by an investigator from behind one-way mirror glass during the experiment.

**FIGURE 1:**
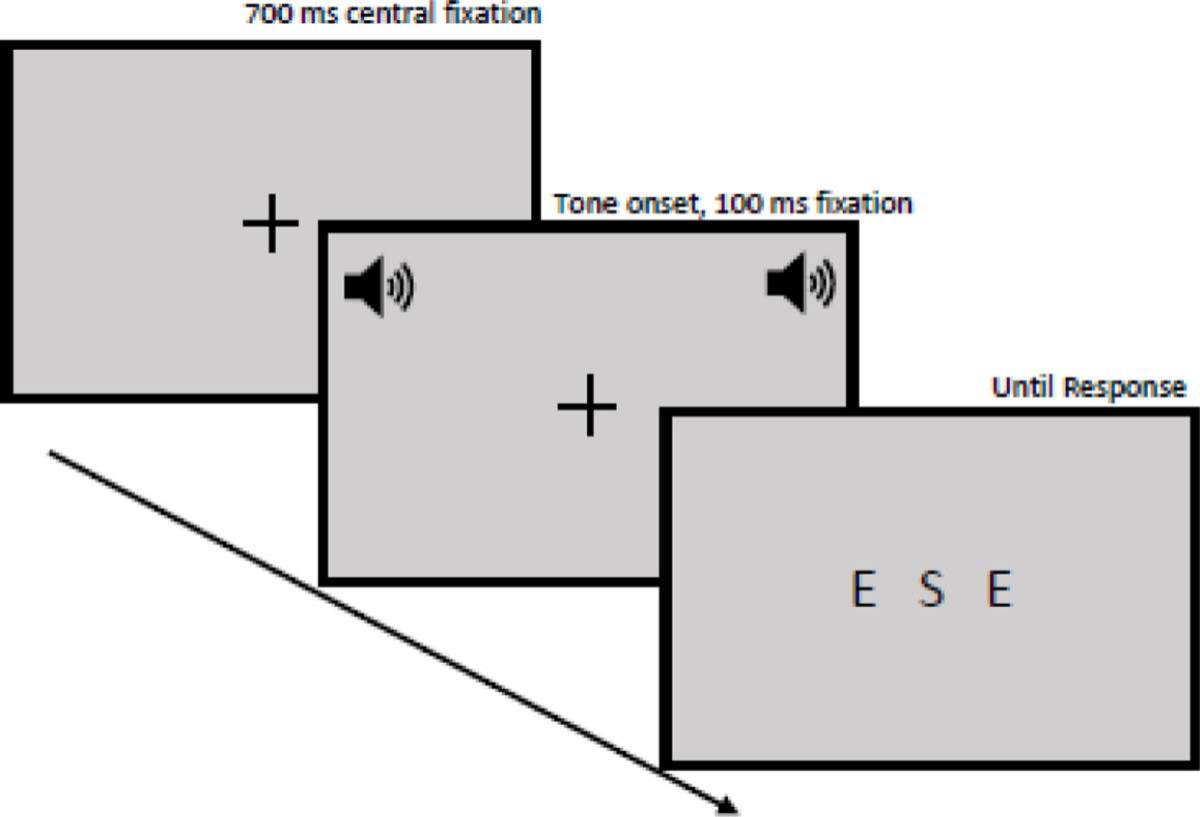
Example of the time-course for a sound-present trial with an incongruent flanker. Sound-absent trials had the same timing and visual displays. Letter mapping changed between Experiments 1 and 2, although timing and display layout remained the same.

The task employed is a classic flanker task consisting of three letters (see Fig. 1). The central letter was the target and was always presented with a flanker letter on each side. The identity of the flanker letters could match or differ from the identity of the central letter. Both left and right flanker letters had the same identity on a given trial. The flankers were 2° away from the central letter in half the trials and 4° away in the other half. All letters were approximately .93° × 1.15 ° and appeared in black on a mid-gray background. The letter display remained on the screen until response. In 50% of the trials (sound-present), a 20 ms sine tone (500 Hz) was played through both sides of the headphones at 65 dB 100 ms before the onset of the letter display. This timing was chosen according to previous research showing auditory tones to be most effective when presented between 100 and 500 ms prior to the onset of the visual stimuli (*e.g*., Fuentes & Campoy, 2008). There was no tone presented in the remaining 50% of trials (sound-absent).

#### Design

Each trial display consisted of some combination of the letters S, C, E, and H. The target letters S and C were assigned to the top left gamepad button and E and H to the top right gamepad button. There were three congruency conditions. The *stimuli-congruent* condition was when the target had the same identity and same button response as the flanker letters (*e.g.,* S S S). The *response-congruent* condition was when the target had a different identity from the flanker letters but the same button response (*e.g*., C S C). The *incongruent* condition was when the target had a different identity and button response from the flanker letters (*e.g*., E S E). These three conditions were crossed with the sound-present vs. sound-absent manipulation. This yielded 24 trials of each congruency condition coupled with the tone and 24 trials with no tone per block of 144 trials. In half of these 24 trials, flankers appeared 2° from the target letter and in the other half they appeared at 4° from the target letter. All trial types were randomly intermixed. There were 4 blocks, resulting in a total of 96 trials for each critical condition. Each block also included two breaks when the participant was prompted to relax their eyes.

#### Procedure

Before starting the experiment, participants received verbal instructions about the task accompanied by a printout showing example stimuli. Participants were instructed to respond only to the middle letter identity and ignore the other letters. Participants were also instructed that sounds were irrelevant and to focus on the letter task. Making responses as quickly and accurately as possible was emphasized. Figure 1 shows an example of trial events. On each trial, a fixation cross appeared and participants were required to fixate within a 0.5° radius for 400 ms before the trial progressed to ensure central fixation. Then, 300 ms after fixation was achieved, the tone occurred on sound-present trials. Finally, 100 ms after the onset of the tone, the letter display was presented with the central letter replacing the fixation cross. On trials without a tone, timing was the same simply minus audible tone presentation. After completing the experiment, participants were debriefed and granted course credit.

#### Analysis

For each participant, trials with reaction times outside of 3 standard deviations for that condition were removed. This resulted in the removal of 1.5% of trials overall. Performance as a function of eccentricity is shown in Table 1. There was no main effect of the flanker eccentricity manipulation on reaction time (*F*(1,14) = 3.43, *p* = 0.09, η_p_^2^ = 0.19). RTs seemed to be reduced with increased eccentricity mainly for the incongruent conditions, although this interaction of eccentricity and flanker type did not reach significance (F(2,28) = 3.09, p = 0.06, η_p_^2^ = 0.18). Previous literature has shown varying compatibility effects on RT to a central target when distractors are between eccentricities of 1 to 5° (*i.e.,* Egeth, 1977; Gatti & Egeth, 1978. On the other hand, Eriksen and Eriksen (1974) found no changes for distractors further than 1° in the periphery. While our results suggest that separation may have some effect on flanker processing, critically there were no significant interactions involving eccentricity and sound presence (all *F*s < 1). Since these null results prevent further conclusions about sound from being drawn, all further analyses collapse over this eccentricity manipulation.

### Results

#### Accuracy

**FIGURE 2:**
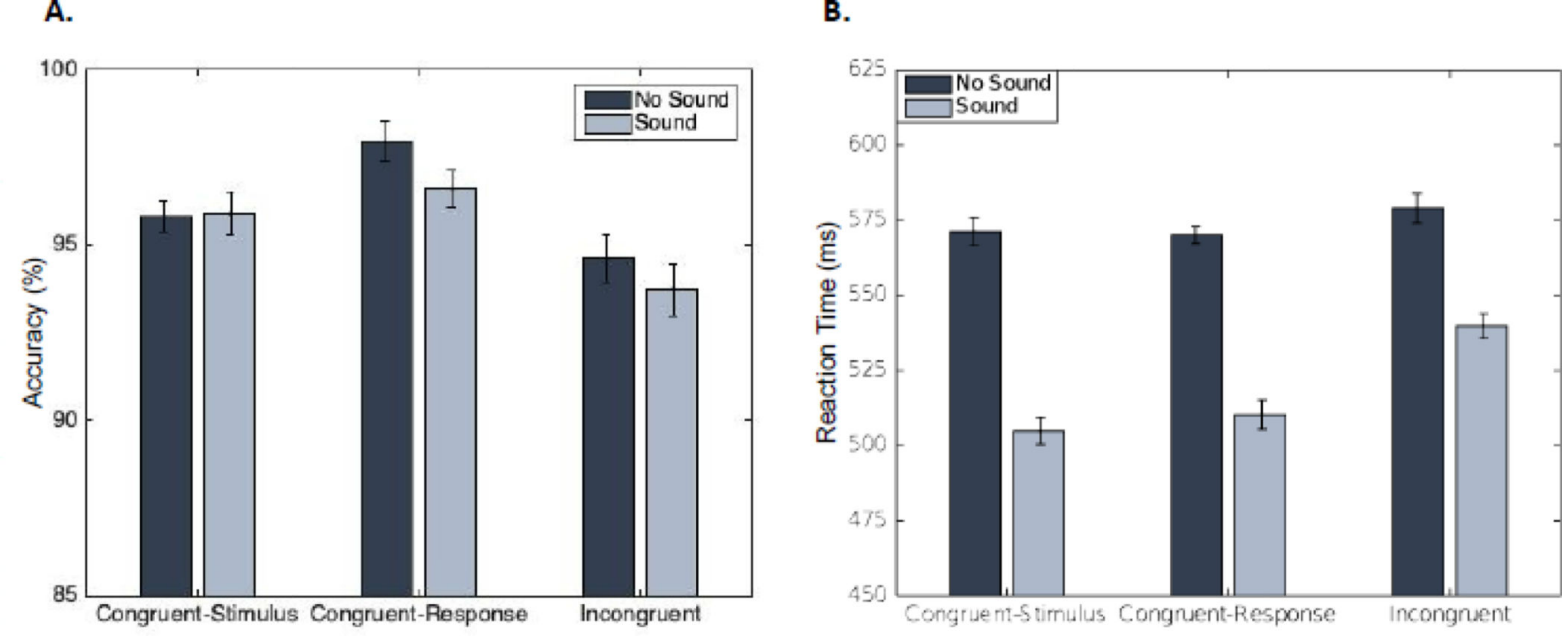
Experiment 1 results. A) Mean accuracy for sound-present and sound-absent conditions. Error bars here and subsequently are condition-specific within-subject 95% confidence intervals (Morey, 2008). B) Mean reaction time for sound-present and sound-absent conditions.

Accuracy was high with participants completing 95% of trials correctly overall (see Fig. 2a). There was a significant effect of flanker type (*F*(2,28) = 13.7, *p* < 0.001, η_p_^2^ = 0.49). Consistent with previous flanker studies, this was driven by more errors in the incongruent condition compared to the other conditions (p < 0.001, Bonferroni-corrected). However, there was no main effect of sound presence (*F*(2,28) = 2.14, *p* = 0.17, η_p_^2^ = 0.13) or interaction of flanker type and sound (*F*(2,28) = 1.48, *p* = 0.25, η_p_^2^ = 0.10). Therefore, only correct trials were further analyzed.

#### Reaction time

RT is reported for each flanker type and sound presence condition in Figure 2b. The tone acted as an alert that decreased RT across conditions, supported by a significant main effect of sound presence (*F* (1,14) = 181.4, *p* < 0.001, η_p_^2^ = 0.93). The congruency manipulation resulted in the expected pattern, with incongruent trials having slower RTs. Consistent with this, there was a main effect of flanker type (*F*(2,28) = 20.9, *p* < 0.001, η_p_^2^ =0.60). Importantly for the purposes of this experiment, the flanker type effect was mediated by an interaction with sound presence (*F*(2,28) = 8.67, *p* = 0.001, η_p_^2^ = 0.38).

Planned comparisons were done to examine the differential speeding by sound, with a Bonferroni-corrected alpha value of 0.017 used to assess significance. The two types of congruent flanker conditions were both speeded by the presence of a tone (67 ms for congruent-stimulus and 60 ms for congruent-response), although the magnitude of this facilitation did not differ significantly (*t*(14) = 1.12, *p* = 0.28, d = 0.29). On the contrary, the incongruent flanker condition was speeded by the tone only 40 ms. This facilitation was significantly smaller than that of the congruent-stimulus condition (*t*(14) = 3.34, *p* =0.005, d = 0.85) and also the congruent-response condition (*t*(14) = 3.31, *p* = 0.005, d = 0.86).

To better understand the influence of sound on response time, standard deviation of response times in each condition were calculated. For the sound present condition, RT standard deviations were 104.5, 103.8, and 116.6 ms for congruent-stimulus, congruent-response, and incongruent flanker conditions respectively. On trials with no sound, these standard deviations were 114.5, 110.6, and 116.1 ms. An ANOVA of this measure showed a significant main effect of sound, such that its presence reduced RT variability (*F*(1,14) = 5.3, p = 0.04, ηp2 =0.28). There was also a main effect of flanker type (*F*(2,28) = 5.1, p = 0.01, ηp2 =0.27), driven by significantly higher variance for incongruent compared to the congruent-stimuli (p = 0.01, Bonferroni-corrected). However, the interaction was not significant (*F*(2,28) = 1.37, p = 0.27, ηp2 =0.09).

### Discussion

The results indicate that the auditory tone presented before the flanker display led to faster RT overall and effectively serves as an alert. To better understand effects on RT, we examined their variability within each condition. As previously found by Wu *et al.* (2011), there was higher variance in RT for incongruent flankers conditions. Moreover, the presence of a sound reduced reaction time variability, which may suggest it better enabled participants to coordinate their response with the onset of the stimulus display. Critically, the tone also differentially affected RTs with regard to flanker type, such that RTs are speeded less for the incongruent condition compared to the congruent conditions. These results are consistent with the idea that flanking visual distractors are processed more following an auditory tone.

The design of Experiment 1 produced two types of congruent conditions due to the assignment of multiple letters to each response button. Central and flanking letters could have the same identity and same response (*stimuli-congruent*) or have different identities but still have the same associated response (*response-congruent*). Although no significant differences of the effect of sound were found between these conditions, the results do clearly show a difference between the congruent and incongruent. In a second experiment, we sought to further investigate how response-association may relate to this sound facilitation.

## Experiment 2

Experiment 2 aimed to further investigate if response-conflict caused by the incongruent flankers was the factor that reduced response speeding by the tone. To do so, a neutral condition utilizing a flanker letter that never appeared as the target and had no assigned response was included. Furthermore, the congruent condition was exclusively comprised of displays in which the flanker letters match the central letter identity (previously *stimuli-congruent* condition). These design changes allowed for analysis of the effect of the tone across three clearly defined, discrete flanker conditions.

Neutral flankers do not cause RT slowing compared to a no-flanker condition when sufficiently spaced from the central target (Eriksen, 1995), and thus could help isolate effects of the sound. If a nonspatial sound generally increases the integration of flanker response association, RT for the congruent flankers should be faster than that for the neutral flankers. This would represent alerting benefits plus facilitation from increased activation of congruent flanker response-association. Likewise, if a nonspatial sound generally increases the integration of flanker response association, trials with incongruent flankers would be expected to show the least benefit. This would represent alerting benefits negated by increased response interference from the incongruent flankers. Such findings of facilitation for congruent relative to neutral and slowing for incongruent relative to neutral would support an account of greater attention to flankers (Kusnir *et al.*, 2011), as well as an account of greater flanker response-association activation (Fischer, Plessow, & Kiesel, 2012). However, if the RT slowing caused by a nonspatial sound is specifically due to the increased presence of response conflict, we might expect that facilitation by the sound would be the same for congruent and neutral, and only less for the incongruent condition. This would be consistent with previous work showing that flanker effects are often largely due to interference from incongruent flankers, and not facilitation from congruent flankers (Schaffer & La Berge, 1979).

Experiment 2 also provided an opportunity to replicate our previous finding that interference from the incongruent flanker is increased by an auditory tone compared to the congruent condition, resulting in less RT benefit. Furthermore, this would enable us to replicate the finding that the auditory tone led to reduced RT variability. Thus, we predicted that RT would be speeded overall and be less variable after hearing an auditory tone due to generalized alerting, although less so for incongruent trials due to enhanced processing of conflicting response-mapping in the flanking visual distractors.

### Methods

#### Participants

A total of 16 participants (mean age 20.4, 12 female) completed Experiment 2 and received course credit. Our goal was again an N of 15, but this was surpassed due to an extra participant enrolling and no technical difficulties. All participants were included in the analysis reported below.

#### Apparatus and Stimuli

All apparatus and stimuli used for Experiment 2 were identical to those used in Experiment 1.

#### Design

In Experiment 2, each flanker task trial was comprised of some combination of the letters S, E, and O. For each participant, these letters were randomly assigned such that two were given unique responses and the third became a neutral letter with no associated response. Each letter combination was equally likely, yielding three types of congruency: congruent (*e.g.,* S S S), neutral (central letter has different identity from flankers which have no assigned response, *e.g.,* O S O), and incongruent (central letter has different identity and button response from the flankers, *e.g.*, E S E). Half of flankers appeared at 2° from fixation and half at 4° from fixation, evenly distributed between the three congruency types. As before, half of the trials from each stimulus type had an auditory tone. All conditions were randomly intermixed. There were 144 trials per block and 4 blocks with breaks as before. This yielded a total of 96 trials for each congruency condition with sound present and sound absent.

#### Procedure and Analysis

The general experimental procedure was the same as that used in Experiment 1. As before, trials with RTs outside of 3 standard deviations for that specific condition were removed. On average, this resulted in the removal of 1.5% of trials. Table 2 provides performance measures separately for each eccentricity. There was no main effect of the manipulation of flanker eccentricity on RT (*F*(1,15) = 2.2, *p* = 0.16). Following from the trend in Experiment 1, there was a significant interaction of flanker separation and flanker type, *F*(2,30) = 3.5, p = 0.04, driven by faster RTs when incongruent flankers were far rather than near. Again, eccentricity did not significantly interact with sound (all *p*s > 0.28). Therefore, all analyses reported collapse over this manipulation.

### Results

#### Accuracy

**FIGURE 3:**
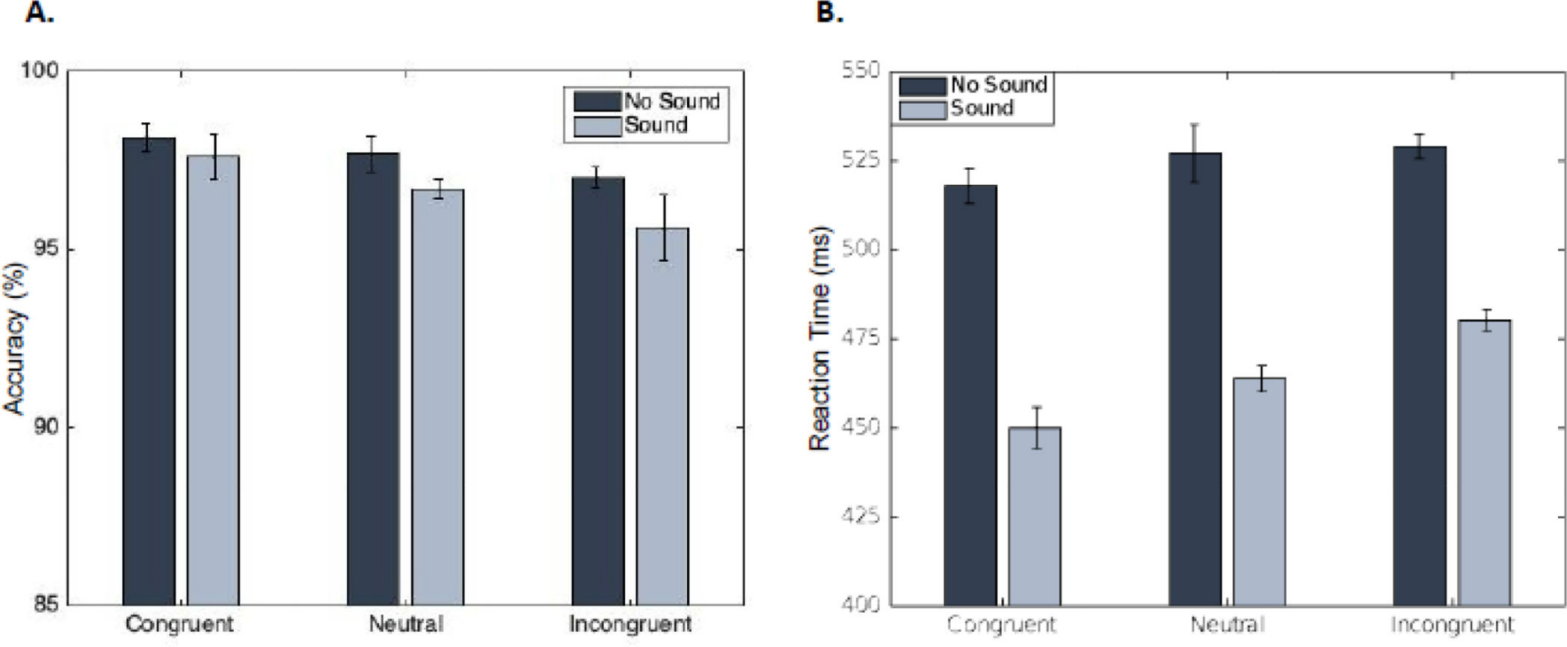
Experiment 2 results. A) Mean accuracy for sound-present and sound-absent conditions. B) Reaction times for sound-present and sound-absent conditions.

Overall accuracy was high with an average of 97.1% correct trials (see Fig. 3a). As in Experiment 1, there was a main effect of flanker type (*F*(2,30) = 4.06, *p* = 0.03, η_p_^2^ = 0.21), which was due to more errors for incongruent than the other conditions (p<0.001, Bonferroni-corrected). There was also a significant effect of sound presence (*F*(1,15) = 6.83, *p* = 0.02, η_p_^2^ = 0.31). On average, there was a 1% decrease in accuracy for the sound-present condition. There was however no significant interaction between sound and flanker type (*F*(2,30) = 0.58, *p* = 0.57, η_p_^2^ = 0.04), so as in Experiment 1, only correct trials were further analyzed.

#### Reaction Time

As shown in Figure 3b, RTs were speeded by the tone across flanker conditions (*F*(1,15) = 135.2, *p* < 0.001, η_p_^2^ = 0.90 ) and there was a main effect of flanker congruency (*F*(2,30) = 10.18, *p* < 0.001, η_p_^2^ = 0.40). As before, flanker congruency significantly interacted with sound presence (*F*(2,30) = 4.8 *p* = 0.02, η_p_^2^ = 0.24). To examine these results in a way comparable to Experiment 1, an additional ANOVA only including the congruent and incongruent flanker conditions and sound presence was conducted. As before, there were significant main effects of sound (*F*(1, 15) = 165.58, *p* < 0.001, η_p_^2^ = 0.92) and flanker type (*F*(1,15) = 42.29, *p* < 0.001, η_p_^2^ = 0.74). Again, there was a significant interaction (*F*(1,15) = 8.82, *p* = 0.01, η_p_^2^ = 0.37).

Given our goal to understand the influence of sound across conditions, as done in Experiment 1, difference scores between sound and no sound were calculated to better understand the nature of this interaction. A one-way ANOVA on these difference scores showed a main effect of flanker type, *F*(1,15) = 4.8, p = 0.02, η_p_^2^ = 0.24. Post-hoc comparison used a Bonferroni-corrected alpha of 0.017 (0.05/3) to determine significance. This showed that the effect of sound on congruent trials was significantly larger than on incongruent trials (68 ms vs. 49 ms, p = 0.01), which replicates the finding from Experiment 1. The difference in the effect of sound between incongruent and neutral did not reach significance (49 ms vs. 63 ms, p = 0.06). Finally, there was no significant difference in the effect of sound between the congruent and neutral flankers (68 ms vs 63 ms, p = 0.41). Bayes factor analysis (Rouder, Speckman, Sun, Morey, & Iverson, 2009) using the default scaling factor of 0.707 indicated it was 2.9 times more likely to arise from chance than a true difference between these conditions.

As in Experiment 1, standard deviation of RTs in each condition were submitted to an ANOVA to examine variability. There was a significant reduction in RT variability with sound, (*F*(1,15) = 19.7, p < 0.001, η_p_^2^ =0.57). However, unlike in Experiment 1, there was no significant effect of flanker type (*F*(1,15) = 0.80, p = 0.46, η_p_^2^ =0.05). As before, there was no interaction of sound and flanker type (*F*(2,30) = 2.42, p = 0.11, η_p_^2^ =0.14).

### Discussion

Experiment 2 again showed the general alerting effect associated with the presentation of an irrelevant tone, with faster RTs overall and less response variability in the sound-present condition. As in Experiment 1, the tone led to less of an RT benefit on incongruent trials compared to congruent trials. These results again support the idea that a non-spatial auditory tone changes the processing of task-irrelevant distractors in the visual field.

The addition of the neutral condition did not provide a definitive answer about differential costs vs benefit. Bayes analysis suggested that the RT benefit caused by sound for congruent and neutral flanker types were likely not different from each other. Although not conclusive, this null effect is consistent with previous work showing that more generally, even without regard to sound, flankers that share a response with a target do not have much effect on response time (Eriksen, Goettl, James, & Fournier,1989; Schaffer & La Berge, 1979). There was also a strong trend for more RT speeding in the neutral condition compared to the incongruent condition. Taken together, these results suggest that sound may specifically lead to additional interference from incongruent flankers. However, future work will be needed to better isolate if the presence of response conflict is responsible for the increased RT interference after a tone.

### General Discussion

The goal of the two experiments described here was to better understand how irrelevant, non-spatial auditory tones influence the processing of irrelevant visual distractors in a flanker task. Previous results have shown a simple auditory tone presented before a visual task reduces RTs overall (Bertelson, 1967; Niemi & Näätänen, 1981). The current results show that the tone led to overall RT speeding and variability reduction across conditions. The finding that the preceding auditory tone reduced response variability is noteworthy, as our literature search did not find other basic studies of alerting reporting this effect. Understanding these changes is of particular interest, as recent work has revealed intraindividual response variability as an important variant in ADHD and autism (*e.g.* Karalunas, Geurts, Konrad, Bender, & Nigg, 2014).

**FIGURE 4:**
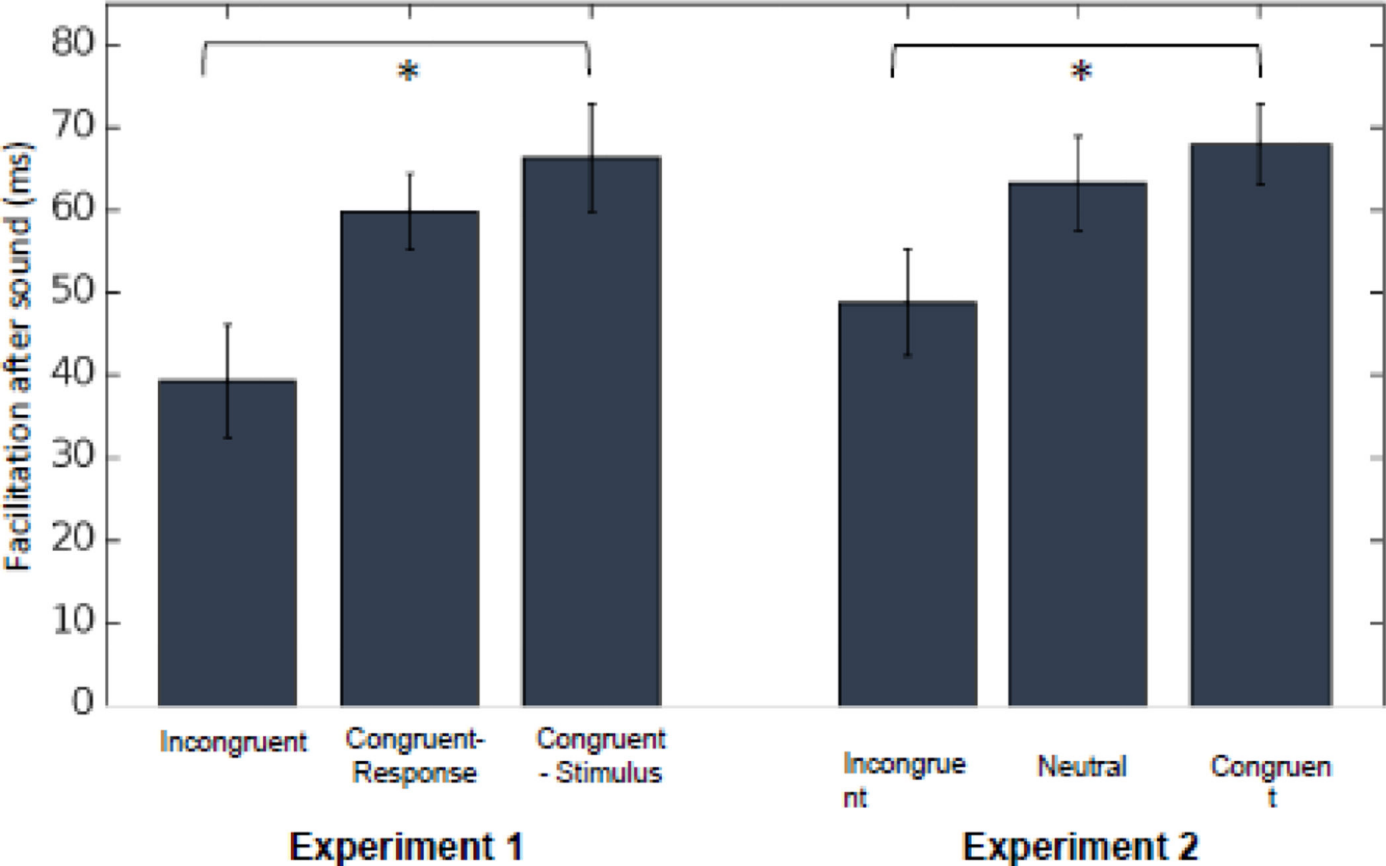
Facilitation on sound-present vs. sound-absent conditions for each flanker type across Expt. 1 and Expt. 2.

A critical finding was that, in two experiments, the facilitation of response caused by the tone was not equivalent across flanker types. Thus, the current results provide evidence that a sound changes the flow of information processing for stimuli presented shortly after its occurrence. The occurrence of an irrelevant tone before the flanker task led to less RT speeding in the presence of incongruent flankers than it did for other flanker types (see Fig. 4 for a summary of results). This suggests that the sound caused general alerting benefits across conditions, combined with some increased interference that reduced these benefits for incongruent flankers.

As described in the introduction, this could be because a nonspatial sound increased attentional processing of the flankers. Weinbach and Henik (2012) also suggested that an auditory tone may change spatial attention using a longer delay between sound and display (500 ms) and arrow stimuli with a more automatic response mapping. Together, the current results and those of Weinbach and Henik (2012) support the hypothesis that nonspatial task-irrelevant sounds may enhance attention to distractors at locations flanking the target. Why might such a crossmodal effect exist? One may speculate that the onset of a nonspatial sound might enhance sensitivity to other objects in the visual field in an attempt to locate the source of that sound. Interestingly, hearing-impaired individuals show increased peripheral spatial attention to distractors appearing even 25° in the periphery (Hong Lore & Song, 1991), which could reflect an adaptive compensation for the lack of spatial broadening triggered by sound detection.

While the current results show that a nonspatial sound altered flanker effects, our manipulation of flanker eccentricity did not interact with the presence of a sound in either experiment. This could be because our choice of eccentricities did not lead to large spatial effects, or that these eccentricities did not span a range differentially modulated by sound. Therefore, our current data cannot definitively speak to whether alerts modulate the breadth of the attentional window, although others have come to the conclusion that they do not. Seibold (2018) used a paradigm in which flanker trials were intermixed with less frequent go/no go probe task trials, with probes presented at different eccentricities to judge the scope of visual attention. While an auditory alert increased congruency effects in the main task, RTs for the probes were facilitated across all flanking locations. A similar conclusion that alerting signals do not increase the size of attentional focus was drawn by Schneider (2018), although a visual cue was used as an alert in his experiments and thus they do not reflect the same crossmodal effects.

The increased flanker effects that we see after a nonspatial sound could also be explained by nonspatial changes in attention such as improved efficiency of attention to the central target. Such an account may also explain the reduction in RT variability we saw in both Experiment 1 and Experiment 2. This is supported by the work of Cho, Lien, and Proctor (2006), which used a Stroop paradigm with a centrally-presented target and a peripheral distractor, a design similar to our own. In a critical experiment, a manipulation of display duration suggested that the distractor was not processed to the level of meaning automatically, but rather was processed as a result of a shift of attention. In our current experiment, if the sound facilitated attentional processing of the central target, attention may have been more available to process the flankers and their response association before a response was generated.

According to the previously-explored accounts of our results, the sound in some way facilitated attentional processing of flankers, either through the increased attention to peripheral flankers or through improved efficiency in target processing itself. These accounts suggest that increased attention to the flankers may have occurred at a perceptual stage, leading to a subsequent amplified processing of response-associations. However, notably, models such as that of Eriksen & Schultz (1979) propose that flanker processing proceeds in parallel to the level of response activation, independent of the allocation of attention. Therefore, it is possible that the current effects represent the effect of the nonspatial sound interacting with the response selection process more directly. In Experiment 2, our results suggested that differential RT speeding by sound may be more driven by slowing from incongruent flankers. The sound may lead to faster linking of flankers with response associations, thus increasing competition and slowing RT (Fischer *et al*., 2012). The activation of response-associations by incongruent flankers demands the need for conflict-monitoring to effectively engage inhibition of task-irrelevant response (Botvinick, Braver, Barch, Carter, & Cohen, 2001). Thus, less facilitation for incongruent flanker trials after a sound could also be due to the reduced availability of such control.

The current results clearly show that nonspatial sounds do not only act as generalized alerts, but also alter the perceptual or response-level processing of flankers. Future work will be needed to better understand the specific mechanisms by which such nonspatial sounds alter subsequent visual processing. However, it is clear that, even when task-irrelevant, auditory tones influence information flow about visual stimuli in such a way to significantly alter behavior. The type of crossmodal interaction reported in the current work is an example of how information processed by one system may have short-term effects on processing of information from other modalities as well. Without question, perceptual and cognitive processing must be dynamically responsive to the rich multisensory environment to cope with the challenges of everyday life.

## Figures and Tables

**Table 1:** Experiment 1 mean RT and accuracies across flanker eccentricity conditions

Flanker Type	No Sound		Sound	

	2 deg Distance RT, ms (Acc %)	4 deg Distance RT, ms (Acc %)	2 deg Distance RT, ms (Acc %)	4 deg Distance RT, ms (Acc %)
Stimuli-Congruent	575.1 (95.9)	568.6 (95.8)	505.3 (96.2)	507.8 (95.8)
Response-Congruent	573.4 (98.2)	570.5 (97.8)	509.6 (96.7)	510.6 (96.5)
Incongruent	586.2 (94.3)	572.3 (94.9)	547.5 (92.9)	533.6 (94.6)

Expt 1 results shown as a function of flanker eccentricity across conditions. Mean RTs for correct trials are shown with accuracy in parentheses

**Table 2:** Experiment 2 mean RT and accuracies across flanker eccentricity conditions

Flanker type	No Sound		Sound	

	2 deg Distance RT, ms (Acc %)	4 deg Distance RT, ms (Acc %)	2 deg Distance RT, ms (Acc %)	4 deg Distance RT, ms (Acc %)
Congruent	516.3 (97.5)	518.1 (98.7)	448.8 (98.7)	452.7 (96.7)
Neutral	525.4 (96.9)	529.3 (98.4)	466.5 (96.9 )	462.8 (96.4)
Incongruent	536.4 (96.0)	523.4 (98.0)	490.5 (94.9)	468.2 (96.3)

Expt 2 results shown as a function of flanker eccentricity across conditions. Mean RTs for correct trials are shown with accuracy in parentheses.

## Footnotes

1 In an exploratory analysis suggested by reviewers, we did find a significant reduction in eye movements between test onset and response after the presentation of a sound in Expt. 2, F(1,15) = 4.4, p = 0.05, ηp2 = 0.23. This is consistent with other work showing sounds may facilitate search by extending fixation duration (Zou, Müller, & Shi, 2012).
